# Expression, Purification, Refolding, and Characterization of a *Neverland* Protein From *Caenorhabditis elegans*

**DOI:** 10.3389/fbioe.2020.593041

**Published:** 2020-10-21

**Authors:** Shuhong Mao, Zhan Song, Mian Wu, Xiaorui Wang, Fuping Lu, Hui-Min Qin

**Affiliations:** ^1^Key Laboratory of Industrial Fermentation Microbiology, Ministry of Education, Tianjin University of Science and Technology, Tianjin, China; ^2^Tianjin Key Laboratory of Industrial Microbiology, Tianjin University of Science and Technology, Tianjin, China; ^3^College of Biotechnology, Tianjin University of Science and Technology, Tianjin, China; ^4^National Engineering Laboratory for Industrial Enzymes, Tianjin, China

**Keywords:** *neverland*, maltose-binding protein, refolding, soluble expression, structural model-3-

## Abstract

Steroid hormones that serve as vital compounds are necessary for the development and metabolism of a variety of organisms. The *neverland* (NVD) family genes encode the conserved Rieske-type oxygenases, which are accountable for the dehydrogenation during the synthesis and regulation of steroid hormones. However, the His-tagged NVD protein from *Caenorhabditis elegans* expresses as inclusion bodies in *Escherichia coli* BL21 (DE3). This bottleneck can be solved through refolding by urea or the introduction of a maltose-binding protein (MBP) tag at the *N*-terminus. Through further research on purification after the introduction of a MBP tag at the *N*-terminus, the CD measurement and fluorescence-based thermal shift assay indicated that MBP was favorable for the NVD proteins’ solubility and stability, which may be beneficial for the large-scale manufacture of NVD protein for further research. The structural model contained the Rieske [2Fe–2S] domain and non-heme iron-binding motif, which were similar to 3-ketosteroid 9 α-hydroxylase.

## Introduction

Sterol derivatives mediate a wide range of growth, development, and evolution in most living species (Thummel and Chory, 2002). In insects, the steroid hormone ecdysone plays an essential role in the developmental transitions and egg production (Huang et al., 2008). The flies could not reach the adult stage when the synthesis for lathosterol was disabled by shutting down the NVD gene using the RNA interference (RNAi) *in vivo*. This matter can be solved by supplementing the standard food or lathosterol on time (Lang et al., 2012). The sterol metabolites also had many important properties, mostly related to the biosynthesis and regulation of amino acids and vitamins (Romero et al., 2005), which are involved in cholesterol homeostasis and synthesis of vitamin D_3_.

The metabolites of vitamin D_3_ (cholecalciferol) have raised a great concern due to its biological effects and physiological properties, such as calcium metabolism and phosphate homeostasis, regulation of immune responses, promotion of insulin secretion, and stimulation of cell proliferation and differentiation (Di Rosa et al., 2011). The vitamin D_3_ was synthesized in humans, and most of the vertebrate animals on the skin, in which the 7-dehydroxycholesterol (7-DHC) was converted into pre-vitamin D_3_ via ultraviolet (UV) irradiation at wavelengths of 290–320 nm and, meanwhile, followed by a thermal isomerization to form vitamin D_3_ spontaneously (Tripkovic et al., 2012). The high incidence of renal bone disease, osteomalacia and osteoporosis, was reported to be associated with the malabsorption of calcium, which is caused by a deficiency of vitamin D_3_ (Kanis, 1999). Compared with the chemosynthesis of 7-DHC using cholesterol (Dugas and Brunel, 2006), the bioconversion of cholesterol into 7-DHC has attracted more attention with regioselectivity and no pollution for the environment (Yoshiyama et al., 2006; Wollam et al., 2011; Yoshiyama-Yanagawa et al., 2011; Lang et al., 2012). The reaction can be catalyzed by the evolutionarily conserved Rieske-domain oxygenase *neverland* (NVD), which contains a Rieske [2Fe–2S] cluster binding domain to function as an electron acceptor and electron transfer, and a highly conserved non-heme iron-binding center as a catalytic domain (Yoshiyama et al., 2006). Several Rieske-domain oxygenase genes from reptiles, insects, nematodes, and deuterostome species had been reported including *Anolis carolinensis* (protein ID XP_003230725.2), *Anopheles gambiae* (protein ID EAA04927.5) (Holt et al., 2002), *Bombyx mori* (protein ID BAE94192.1) (Yamanaka et al., 2005), *Caenorhabditis elegans* (protein ID CAA98235.2) (Rottiers et al., 2006), *Ciona intestinalis* (protein ID BAK39961.1) (Yoshiyama-Yanagawa et al., 2011), *Drosophila melanogaster* (protein ID ABW08586.1) (Warren et al., 2002), *Danaus plexippus* (protein ID OWR46621.1) (Zhan et al., 2011), *Danio rerio* (protein ID BAK39960.1) (Yoshiyama et al., 2006), *Gallus gallus* (protein ID XP_425346.2) (Yoshiyama et al., 2006), *Hemicentrotus pulcherrimus* (protein ID BAK39963.1) (Yoshiyama-Yanagawa et al., 2011), *Rhodococcus rhodochrous* (*kshA*, protein ID ADY18310.1) (Petrusma et al., 2011), *Spodoptera littoralis* (protein ID ADK56283.1) (Iga et al., 2013), *Pseudomonas fluorescens* (*prnD*, protein ID AAB97507.1) (Lee et al., 2005), *Podarcis muralis* (protein ID XP_028576239.1), *Pseudonaja textilis* (protein ID XP_026568371.1), and *Xenopus laevis* (protein ID BAK39959.1) (Yoshiyama-Yanagawa et al., 2011). The family proteins are an essential regulator of cholesterol metabolism and steroidogenesis.

In addition, NVD from *Caenorhabditis elegans* (CeNVD) were identified in the metabolic pathway of cholesterol, and genetic evidence has demonstrated that the NVD gene plays a vital role in the larval development and adult aging in the ecdysteroid biosynthesis (Rottiers et al., 2006; Yoshiyama-Yanagawa et al., 2011). However, there have been a few reports about the effective heterologous expression and production system of the NVD family proteins *in vitro.* Here, we verified that the NVD protein was expressed as inclusion bodies with His-tag (Zhu et al., 2019b), and a small amount of soluble protein was obtained, even though it was further refolded by urea. Subsequently, we introduced the maltose-binding protein (MBP) to enhance the soluble expression and purification of CeNVD in *Escherichia coli* BL21 (DE3), and the thermostability of CeNVD was also improved.

## Materials and Methods

### Materials

The *neverland* gene from *Caenorhabditis elegans* (CeNVD) was chemically synthesized in pET-28a(+) (Novagen, Madison, WI, United States) vector by GENEWZ (Suzhou, China) after codon was optimized. The DNA fragment of 1,110 bp was PCR amplified using gene-specific primers, which contain the *Eco*RI and *Hin*dIII restriction sites at the 5′- and 3′-terminal, and was cloned into the pMal-c2X (New England Biolabs, Beverly, MA, United States) plasmid vector, which contains an N-terminal MBP-tag and sequence. The *E. coli* BL21 (DE3) (Novagen, Darmstadt, Germany) strain was employed as a heterologous expression host.

### Expression and Purification

The recombinant plasmid was transformed into an *E. coli* BL21 (DE3) strain and grown in a Luria–Bertani (LB) medium supplemented with kanamycin (50 μg/ml) or ampicillin (100 μg/ml) with a shaking of 220 rpm at 37°C. When the optical density at 600 nm (OD_600_) reached 0.6–0.8, 0.5 mM, isopropyl-β-D-thiogalactopyranoside (IPTG) was added to the culture, and the recombinant cells were cultivated with a shaking of 160 rpm at 16°C for 16–18 h to induce the protein expression. After cultivation, the recombinant cells were harvested by centrifugation at 5,000 × *g* for 15 min at 4°C and washed twice with PBS (pH 8.0) (Sun et al., 2019).

In order to purify the CeNVD_pET-28a(+), the washed cells were resuspended in a 30-ml lysis buffer A (20 mM Tris–HCl, 20 mM imidazole, 500 mM NaCl, and 1 mM dithiothreitol, pH 8.0) containing 0.5 mg/ml lysozyme and 1 mM phenylmethanesulfonyl fluoride (PMSF) and disrupted using a sonicator (Sonic Dismembrator Model 100, Pittsburgh, PA, United States) on ice bath for 20 min, the unbroken cells and cell debris were removed by centrifugation at 20,000 × *g* for 30 min at 4°C, the supernatant was applied to a nickel-nitrilotriacetic acid (Ni-NTA) agarose affinity chromatography matrix (QIAGEN, Hilden, Germany), and pre-equilibrated with lysis buffer A. After washing the open column with 10-ml of lysis buffer A extensively, the bound protein was eluted with a 10-ml elution buffer A (20 mM Tris–HCl, 300 mM imidazole, 300 mM NaCl, and 1 mM dithiothreitol, pH 8.0) (Mao et al., 2018).

For purification of the CeNVD_pMal-c2X, the amylose resin was applied to the fixed MBP_CeNVD fusion protein. The unbound protein was washed with lysis buffer B (20 mM Tris–HCl, 500 mM NaCl, 1 mM EDTA, and 1 mM dithiothreitol, pH 8.0), and the target protein was eluted with 10 ml of elution buffer B (20 mM Tris–HCl, 20 mM maltose, 500 mM NaCl, 1 mM EDTA, and 1 mM dithiothreitol, pH 8.0) (Zhu et al., 2019b). Then the protein was further purified by an anion exchange chromatography employing a Resource Q column (column volume: 6 ml, flow rate: 4 ml/min, GE Healthcare, Stockholm, Sweden) on the ÄKTA system (GE Healthcare, Sweden) (Sun et al., 2018). The purified enzyme was eluted with a linear gradient between 0 and 1 M NaCl at a flow rate of 3 ml/min. Subsequently, the MBP tag was digested using a Factor Xa Protease (New England Biolabs, Beverly, MA, United States) at 4°C for 12 h and then loaded on an amylose resin to remove the MBP tag and undigested protein. The flow-through buffer containing the target protein was collected and concentrated for further experiments.

### Western Blot Analysis of CeNVD_pET-28a(+)

After the centrifugation of the disrupted CeNVD_pET-28a(+), the cleared supernatant and precipitant were loaded on SDS–PAGE gels, then all the protein molecules was transferred to a PVDF membrane and blocked in a PBST buffer (PBS pH 8.0, 0.02% Tween-20) containing a 1% bovine serum albumin (BSA) for 2 h, followed by incubation in an anti-His-tag mouse monoclonal antibody (Abcam, Cambridge, United Kingdom), which was diluted in a blocking buffer (PBST pH 8.0, 1% BSA) at the indicated concentrations of 1:5,000 for 12 h at 4°C. After washing with PBST for four times, the membrane was protected from light and incubated with the HRP-conjugated secondary antibody (HRP-conjugated goat anti-mouse IgG, Tiangen Biochemical Technology, Beijing, China) at a dilution of 1:1,000 and room temperature for 2 h. After washing, the target protein was trapped by the HRP-DAB chromogenic substrate kit (Tiangen Biochemical Technology, Beijing, China), and the immunoreactive band was digitally scanned using an Odyssey Infrared Imager (LI-COR Bio-science, Lincoln, NE, United States) (Wan et al., 2016).

### The Refolding of Denatured Protein

The CeNVD_pET-28a(+) cells were collected and resuspended in lysis buffer C (20 mM Tris–HCl, 20 mM imidazole, 500 mM NaCl, 8 M urea, and 1 mM DTT, pH 8.0), and then disrupted and centrifuged as mentioned above. The cleared supernatant containing the denatured enzyme was refolded by sequential dialysis with a gradient descent of urea concentrations (7, 6, 5, 4, 3, 2, 1, 0.5, 0 M), then trapped on a pre-equilibrated Ni-NTA superflow resin (QIAGEN, Hilden, Germany), and washed with lysis buffer D (20 mM Tris–HCl, 20 mM imidazole, 500 mM NaCl, 1 mM dithiothreitol, 0.5 mM GSSG, 3 mM GSH, and 500 mM arginine, pH 8.0). The refolded protein was eluted with elution buffer D (20 mM Tris–HCl, 300 mM imidazole,300 mM NaCl, 0.5 mM GSSG, 3 mM GSH, 500 mM arginine, and 1 mM dithiothreitol, pH 8.0) (Qin et al., 2017a). The protein concentration of each purification step was measured via the BCA protein assay kit (Solarbio, Beijing, China) (Qin et al., 2017b).

### Molecular Mass Determination

The molecular weight of the native CeNVD was measured by a gel filtration chromatography using a Superdex200 Increase 10/300 GL column on the ÄKTA system (GE Healthcare, Sweden) (Zhu et al., 2019a). The target enzyme was eluted with a buffer (20 mM Tris–HCl, 150 mM NaCl, and 1 mM DTT, pH 8.0) at a flow rate of 1 ml/min with aldolase (158 kDa), conalbumin (75 kDa) as calibration proteins (GE Healthcare).

### Circular Dichroism Measurements

The circular dichroism (CD) spectra was determined using a MOS-450 CD spectropolarimeter (Biologic, Claix, Charente, France). The protein sample was loaded into a 1-cm path-length quartz cuvette in which 0.1 mg/ml of protein was dissolved in PBS (pH 8.0), and the CD data were recorded in the far-UV band of 190–250 nm at room temperature for an average of four times scan with a rate of 1 nm/s, a bandwidth of 0.1 nm, and a step resolution of 0.1 nm (Zhu et al., 2019c). Analysis of the protein secondary structure was performed with the program BeStSel^1^ (Micsonai et al., 2015, 2018).

### Fluorescence–Based Thermal Shift Assay

The thermal stability of CeNVD was characterized via the fluorescence-based thermal shift assay using a 48-well assay plate real-time PCR instrument (Bio-Rad, Hercules, CA, United States). Reaction samples were conducted in three replicates that contained 0.4 mg/ml of protein and 100 × SYPRO Orange dye in PBS buffer (pH 8.0). The temperature was increased with a linear gradient of 20–90°C at 0.5°C/30 s, and the minimal value was regarded as the melting temperature (*T*_*m*_) (Mao et al., 2020a).

### Structure Modeling of CeNVD

The three-dimensional (3D) homology model of CeNVD was generated by the SWISS-MODEL (swissmodel.expasy.org/) (Guex and Peitsch, 1997) using a template of 3-ketosteroid 9 α-hydroxylases from *R. rhodochrous* (PDB ID: 4QDF, 2.43 Å) (Penfield et al., 2014), which shared a 30% sequence identity with CeNVD. Then the generated models were visualized and analyzed using the PyMoL software^2^.

## Results and Discussion

### Sequence Alignment and Phylogenetic Analysis

**FIGURE 1:**
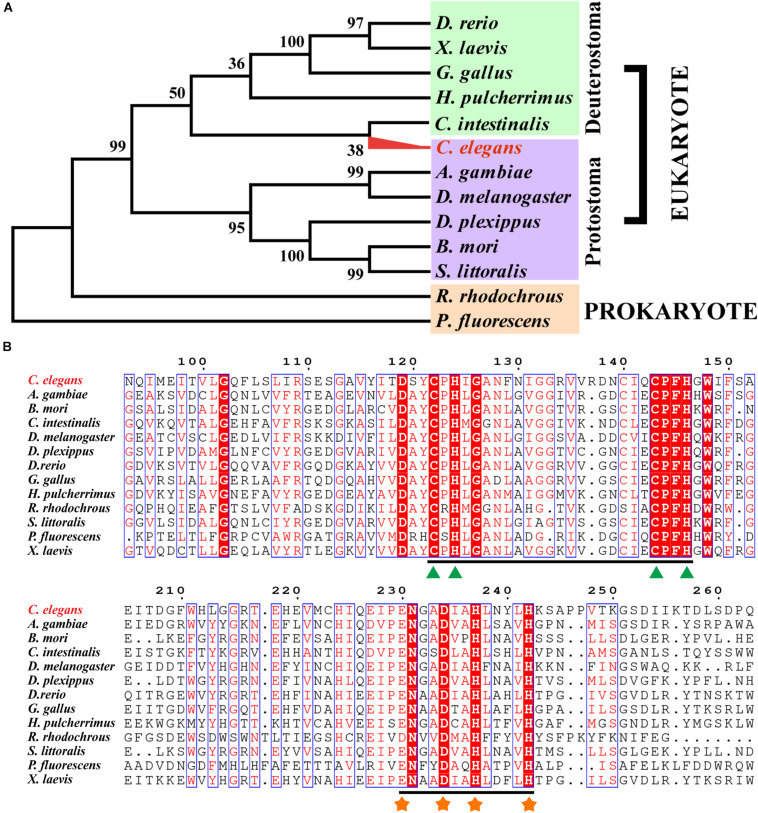
Sequence alignment and phylogenetic tree of *Caenorhabditis elegans* (CeNVD) with family enzymes. **(A)** The phylogenetic analysis of Rieske oxygenases from different species. **(B)** Multiple alignment of CeNVD with other Rieske oxygenases; the green triangle (▲) and orange asterisk (∗) were responsible for the Rieske [2Fe–2S] domain (C-X-H-X16-17-C-X2-H) and non-heme iron-binding motif [Fe(II); E/D-X3-D-X2-H-X4-H], respectively. The alignment was prepared using the program ESPript 3.0 service (http://espript.ibcp.fr/ESPript/ESPript/).

The phylogenetic tree of Rieske oxygenase from various microorganisms revealed that the evolutionary relationship of CeNVD was similar to that of *C. intestinalis* (Figure 1A) with 41.8% amino acid sequence identity. It showed the lower sequence identity of 30.2% with *B. mori*. BLAST and sequence analysis indicated that the NVD from *C. elegans* shared a higher sequence identity with *X. laevis* (45.7%), *D. rerio* (44.9%), *G. gallus* (44.0%), *C. intestinalis* (41.8%), and *A. gambiae* (40.3%). Amino acid sequence alignment and analysis with the homologous proteins displayed that the family proteins contained two evolutionally conserved domains, Rieske [2Fe–2S] domain (C-X-H-X16-17-C-X2-H) and non-heme iron-binding motif [Fe(II); E/D-X3-D-X2-H-X4-H]. The conserved residues in CeNVD contained C122, H124, C143, H146 in the Rieske domain, and E230, D234, H237, H242 in the Fe (II) domain (Figure 1B).

### Heterologous Expression and Purification of CeNVD Recombinant Enzyme

**FIGURE 2:**
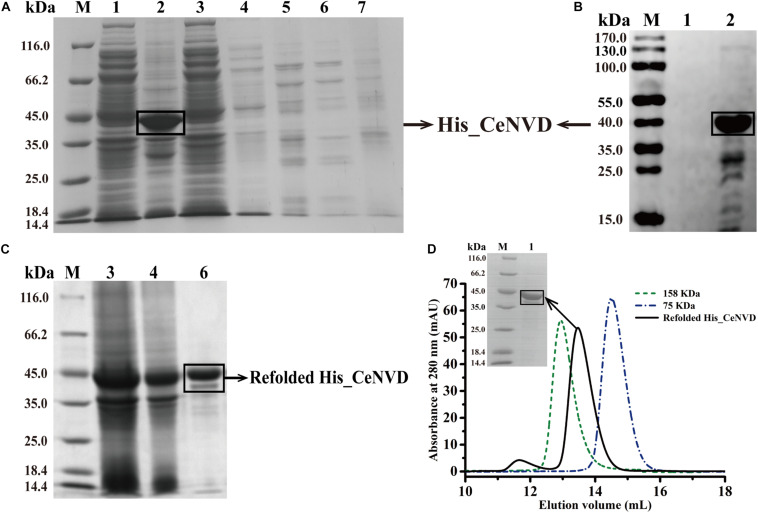
Purification of the CeNVD_pET28a(+) by nickel-nitrilotriacetic acid (Ni-NTA) affinity chromatography. **(A)** SDS-PAGE analysis of CeNVD_pET28a(+). **(B)** Western blot analysis of CeNVD_pET-28a(+). **(C)** SDS-PAGE analysis of the denatured and refolded protein. **(D)** Size-exclusion chromatography of the refolding protein, aldolase (158 kDa), conalbumin (75 kDa) as reference proteins; Lanes 1: supernatant; 2: sediment; 3: flow-through; 4: wash buffer; 5: resin before eluting; 6: elution buffer; 7: resin after eluting.

**FIGURE 3:**
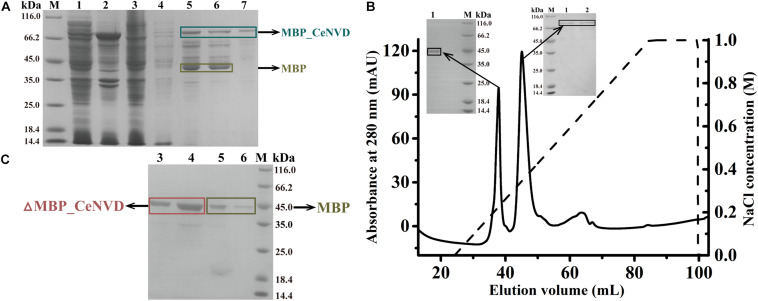
**(A)** Purification of CeNVD_pMal-c2X by the MBP-trap affinity chromatography. **(B)** Purification of CeNVD_pMal-c2X by the anion-exchange. **(C)** Purification of CeNVD by the MBP-trap without the MBP tag. Lanes 1: supernatant; 2: sediment; 3: flow-through; 4: wash buffer; 5: resin before eluting; 6: elution buffer; 7: resin after eluting.

The CeNVD_pET28a(+) was expressed in *E. coli* BL21 (DE3) and purified by the His-trap affinity chromatography. SDS-PAGE and Western blot analysis demonstrated that the target protein appeared as a single band with a molecular mass of approximately 42 kDa, consistent with the calculated molecular weight of 42,800 Da. However, the protein was overwhelmingly expressed as inclusion bodies (Figures 2A,B). Subsequently, the CeNVD gene was cloned and inserted into pMal-c2X. The reconstructed enzyme was overexpressed and purified by a genericmultiple-step purification using an amylose fast flow resin and anion exchange chromatography (Figures 3A,B). The MBP-tag was then digested by a Factor Xa protease and removed by an amylose resin (Figure 3C). The yields and purities of CeNVD for the different purification stages are summarized in Table 1. Finally, 4.1 mg of CeNVD with 91.1% high purity was obtained in 200-ml of cell culture. Therefore, the MBP was advantageous to the soluble expression and purification of CeNVD in *E. coli*, and the purification multiple-step purification method was necessary to obtain highly purified recombinant CeNVD.

**TABLE 1:** Kinetic parameters of KsdD3 WT and mutants toward nine substrates.

**Purification step**	**Total protein (mg)**	**Target protein (mg)**	**Purity (%)**	**Yield (%)**
Supernatant of MBP_CeNVD	72.3	21.4	29.6	100
Eluate from Resource Q	12.6	9.7	77.0	45.3
Flow through from the MBP-trap after digesting the MBP-tag	4.5	4.1	91.1	19.2
Eluate from the Ni-NTA resin after refolding	8.7	6.4	73.6	29.9
Eluate from Superdex200	3.6	3.2	88.9	15.0

*Summary of the yields and enrichment factors of *Caenorhabditis elegans* (CeNVD) for different purification process. The results are based on the cell material from a 200-ml *Escherichia coli* culture.*

### Characterization of the Refolded His_CeNVD

His_CeNVD was expressed as inclusion bodies. Therefore, we added arginine and urea to refold the protein with an extra redox system to improve the refolding yield, such as reduced and oxidized glutathione (GSH and GSSG) (Chen et al., 2016). Here, we used a gradient descent of urea with 500 mM arginine and a redox pair (GSH and GSSG) to facilitate the protein solubilizing and refolding. After dialysis, the refolded His_CeNVD was further purified by an Ni-NTA affinity chromatography (Figure 2C), and the fraction was further analyzed through a gel filtration chromatography, and a single peak was detected at 280 nm. Consistent with the gel filtration chromatography analysis, SDS-PAGE indicated that the refolded His_CeNVD of higher purity (88.9%) (Table 1) was obtained and a trimer state in solution (Figure 2D).

### Characterization of All CeNVD

**FIGURE 4:**
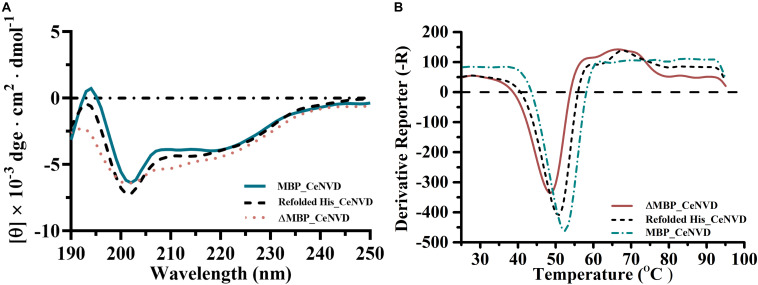
**(A)** The CD spectra and **(B)** melting curves of CeNVD. The experiments were conducted in three replicates, and the data represent the means ± standard deviations.

The secondary structure of the MBP_CeNVD, ΔMBP_CeNVD, and refolded His_CeNVD were measured by CD spectroscopy (Figure 4A). The MBP_CeNVD showed a negative absorption peak centered around 202 nm, and the percentage of α-helix, β-strand, turn, and unordered regions were 9.5%, 24.7%, 8.5%, and 57.3%, respectively. The CD spectrum of the ΔMBP_CeNVD and refolded protein demonstrated a visible increase in α-helix (22.5%, 16.4%) and a slight decrease in turn structures (4.6%, 6.7%) and unstructured regions (45.8% and 51.6%) (Table 2).

**TABLE 2:** Secondary structure assignments (%) of CeNVD determined by circular dichroism (CD) spectroscopy in the wavelength region from 190 to 250 nm.

**Protein**	**α-Helix**	**β-Strand**		**Turn**	**Other**
		**Antiparallel**	**Parallel**		
Refolded His_CeNVD	16.4	11.9	13.4	6.7	51.6
MBP_CeNVD	9.5	8.9	15.8	8.5	57.3
ΔMBP_CeNVD	22.5	17	10.1	4.6	45.8

Fluorescence-based thermal shift assay was used to determine the thermostability of the three types of CeNVD. As shown in Figure 4B, the *T*_*m*_ value of the MBP_CeNVD was higher than the His_CeNVD and ΔMBP-NVD (52.5°C, 50°C, and 48°C), which suggested that the MBP was profitable for the thermostability of CeNVD.

### Structural Analysis of CeNVD

**FIGURE 5:**
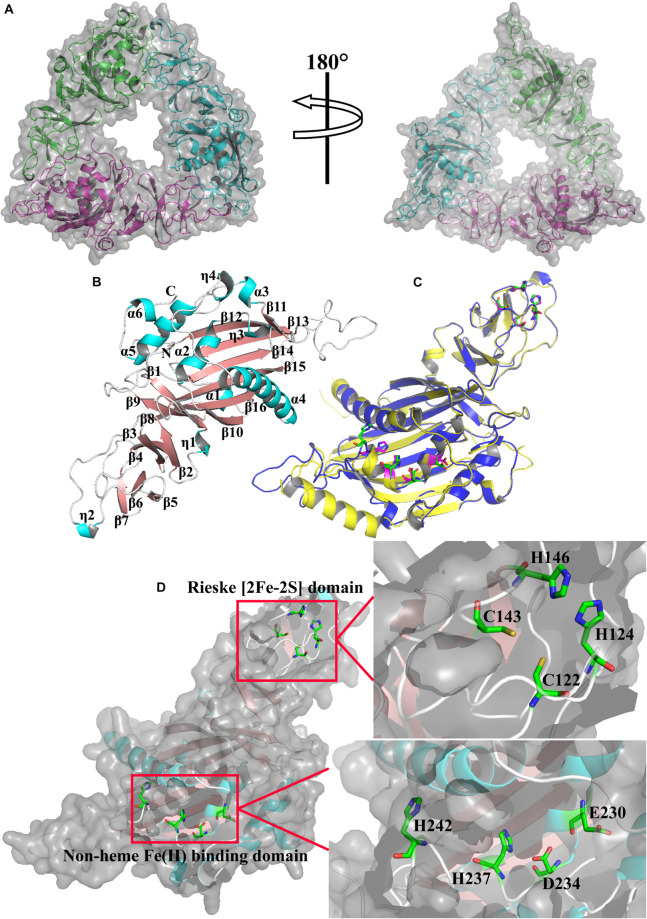
**(A)** The structure model of CeNVD. **(B)** Monomer structure of CeNVD with the α-helices (cyan) and β-strands (salmon) are labeled. **(C)** The superimposed subunits of CeNVD (blue) and the 3-ketosteroid 9 α-hydroxylases from *Rhodococcus rhodochrous* (yellow) with major domains; residues are shown as green and magenta sticks. **(D)** The stereo view of Rieske [2Fe–2S] domain and non-heme Fe(II)-binding domain.

The structure of CeNVD contains six α-helices (α1–α6) and 16 antiparallel β-strands (β1–β16) (Figures 5A,B), which are conserved in the NVD family enzymes. It contains an *N*-terminal Rieske [2Fe-2S] cluster (C122, H124, C143, H146) and followed by the catalytic domain harboring non-heme Fe (II) center (E230, D234, H237, H242). The Rieske cluster was located at strands β4, β5 and β6, β7. As for the Fe (II) center, E230 and D234 are located at α2, H237 is located at η3, and H242 is located on a loop between η3 and β11 (Figure 5D; Penfield et al., 2014). Structural alignment revealed that the structure and residues in the major domains are remarkably similar to that of 3-ketosteroid 9 α-hydroxylases from *R. rhodochrous* (C67, H69, C86, H89) and (D174, D178, H181, H186) (Figure 5C; Mao et al., 2020b).

## Conclusion

Our study provides a methodological approximation for the purification of soluble Rieske domain-containing oxygenase expressed in *E. coli*. We successfully expressed and purified the CeNVD protein in *E. coli* with the soluble formation of refolded His-NVD and MBP-NVD, showing that the MBP tag could increase the soluble expression of CeNVD, which is advantageous for further purification and improvement of thermostability.

## Data Availability Statement

The raw data supporting the conclusions of this article will be made available by the authors, without undue reservation.

## Author Contributions

FL and H-MQ designed the research. SM, ZS, MW, and XW performed the experiments and analyzed the data. H-MQ, SM, and FL wrote the manuscript. All authors read and approved the final manuscript.

## Conflict of Interest

The authors declare that the research was conducted in the absence of any commercial or financial relationships that could be construed as a potential conflict of interest.

## Footnotes

1 http://bestsel.elte.hu

2 http://www.pymol.org
