# Supplementary material for: Expression, Purification, Refolding, and Characterization of a Neverland Protein From Caenorhabditis elegans
Source: Front Bioeng Biotechnol. 2020 Oct 21;8:593041. doi: 10.3389/fbioe.2020.593041 (PMC7609953; doi:10.3389/fbioe.2020.593041)
Supplement: Supplementary file 1 [file Table_1.DOCX]

**Supplementary materials**

**TABLE 1 |** The Genbank accession number of the NVDs used in figure 1 to generate the phylogenetic tree.

| **Organism** | **Genbank accession number** |
| --- | --- |
| *A.carolinensis* | XP_003230725.2 |
| *A.gambiae* | XP_309236.5 |
| *B.mori* | NP_001037626.1 |
| *C.elegans* | NP_505629.2 |
| *C.intestinalis* | NP_001265914.1 |
| *D.melanogaster* | NP_001097670.1 |
| *D.plexippus* | XP_032528372.1 |
| *D.rerio* | NP_001002612.1 |
| *G.gallus* | XP_425346.2 |
| *H.pulcherrimus* | BAK39963.1 |
| *R.erythropolis* | WP_020906621.1 |
| *S.littoralis* | ADK56283.1 |
| *P.fluorescens* | P95483.1 |
| *P.muralis* | XP_028576239.1 |
| *p.textilis* | XP_026568371.1 |
| *X.laevis* | OCT65169.1 |
